# Synthetic propeptide design to enhance the secretion of heterologous proteins by *Saccharomyces cerevisiae*

**DOI:** 10.1002/mbo3.1300

**Published:** 2022-06-09

**Authors:** Ji Sung Cho, Hye Ji Oh, Young Eun Jang, Hyun Jin Kim, Areum Kim, Jong‐Am Song, Eun Jung Lee, Jeewon Lee

**Affiliations:** ^1^ Department of Chemical and Biological Engineering, College of Engineering Korea University Seoul Korea; ^2^ Department of Chemical Engineering, School of Applied Chemical Engineering Kyungpook National University Daegu Korea

**Keywords:** human granulocyte colony‐stimulating factor, *Saccharomyces cerevisiae*, secretion efficiency, synthetic propeptides

## Abstract

Heterologous protein production in *Saccharomyces cerevisiae* is a useful and effective strategy with many advantages, including the secretion of proteins that require posttranslational processing. However, heterologous proteins in *S. cerevisiae* are often secreted at comparatively low levels. To improve the production of the heterologous protein, human granulocyte colony‐stimulating factor (hG‐CSF) in *S. cerevisiae*, a secretion‐enhancing peptide cassette including an hIL‐1β‐derived propeptide, was added and used as a secretion enhancer to alleviate specific bottlenecks in the yeast secretory pathway. The effects of three key parameters—*N*‐glycosylation, net negative charge balance, and glycine‐rich flexible linker—were investigated in batch cultures of *S. cerevisiae*. Using a three‐stage design involving screening, selection, and optimization, the production and secretion of hG‐CSF by *S. cerevisiae* were significantly increased. The amount of extracellular mature hG‐CSF produced by the optimized propeptide after the final stage increased by 190% compared to that of the original propeptide. Although hG‐CSF was used as the model protein in the current study, this strategy applies to the enhanced production of other heterologous proteins, using *S. cerevisiae* as the host.

## Graphical abstract

*Saccharomyces cerevisiae* is widely used for heterologous protein production but often secretes at comparatively low levels. To improve this, a secretion‐enhancing peptide cassette was added to the target protein and the propeptide was optimized using three key parameters: *N*‐glycosylation, net negative charge balance, and glycine‐rich flexible linker. Production of the model protein, the human granulocyte colony‐stimulating factor, was increased by 190% compared to the original propeptide. This stepwise rational design is a promising strategy applicable to other heterologous proteins.

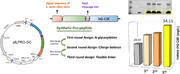

## 1 INTRODUCTION

*Saccharomyces cerevisiae* is one of the most widely used microorganisms for heterologous protein production. This popularity can be attributed to the properties of *S. cerevisiae*, such as rapid growth rate, high cell density cultivation in simplified medium, posttranslational processing, and the ability to secrete heterologous proteins in its native form, which favor its use as an industrial workhorse (Parapouli et al., 2020). In addition, *S. cerevisiae* does not possess any detectable endotoxins and is not pathogenic to humans; hence, it is generally recognized as a safe system for the production of food and healthcare products. Many significant commercial biological products, including insulin, hepatitis B surface antigen, urate oxidase, glucagon, granulocyte‐macrophage colony‐stimulating factor (GM‐CSF), hirudin, and platelet‐derived growth factor are produced using *S. cerevisiae* (Demain & Vaishnav, 2009; Wang et al., 2019).

Among the inherent advantages of *S. cerevisiae*, its ability to secrete proteins is particularly valuable because it simplifies downstream protein recovery and purification processes (Calado et al., 2004; Ilmén et al., 2011). However, the secretory expression of heterologous proteins in *S. cerevisiae* is often subject to bottlenecks, which limit their yield. Factors such as plasmid vector systems (i.e., promoters, leader sequences, and translation signals), cultivation conditions, and target protein properties affect the yield of heterologous proteins. Therefore, many studies have addressed the development of the fermentation process, strain engineering using genetic modification, and the optimization of secretory expression vector systems (Besada‐Lombana & Da Silva, 2019; Hou et al., 2012; Huang et al., 2018; Liu et al., 2012; Mori et al., 2015).

Among these factors, the design and use of signal/leader peptides in vector systems is a relatively simple and highly efficient approach to overcome the issues in secretory expression and is easily applicable to other host strains (Inokuma et al., 2016; Mori et al., 2015; Wirajana et al., 2016). Secretion signals in well‐known secretory proteins, such as acid phosphatase, invertase, and α‐mating factor, have been investigated to improve the production yield of heterologous proteins (Lin‐Cereghino et al., 2013). Human interleukin‐1β (hIL‐1β)‐derived peptide is also a signal/leader peptide capable of inducing protein secretion. Increased secretion occurs from *S. cerevisiae* when it is fused to the *Kluyveromyces lactis* killer toxin leader peptide (Fleer et al., 1991) or the *Candida albicans* glucoamylase leader peptide (Choi et al., 2001; J. Lee et al., 2003; Livi et al., 1991; Song et al., 2002).

However, many heterologous proteins with signal/leader peptides are still secreted at comparatively low levels, even when the transcription/translation level of the heterologous protein is optimized for overexpression. Along with simple protein expression, the secretion of heterologous proteins also depends on many factors, such as the post‐translational translocation of nascent proteins into the endoplasmic reticulum (ER), protein folding and quality control inside the ER, post‐translational glycosylation in the ER and Golgi apparatus, intracellular protein trafficking and sorting, proteolytic degradation, and stress responses to the misfolding or overexpression of proteins. In particular, the process of transport from the ER to the Golgi apparatus in yeast is a rate‐limiting step in the secretory pathway of heterologous proteins (Bao et al., 2018; Hou et al., 2012; Van Zyl et al., 2016). Therefore, the design and optimization of secretion signal/leader peptides for nascent protein folding and the translocation of nascent proteins from the ER to the Golgi apparatus are crucial and may increase the secretion of heterologous proteins.

Human granulocyte colony‐stimulating factor (hG‐CSF) is a hematopoietic cytokine that acts on cells of the neutrophil lineage, causing the proliferation and differentiation of committed precursor cells and the activation of mature neutrophils (Vanz et al., 2008). Since hG‐CSF regulates hematopoietic progenitors and reduces oral toxicity from chemotherapy (Gabrilove et al., 1988), it is often used in intensive chemotherapy for small‐cell lung cancer (Sun et al., 2020; Timmer‐Bonte et al., 2006), chemotherapy‐induced febrile neutropenia (Kuderer et al., 2007; Sung et al., 2004; Whyte et al., 2011), and for the treatment of solid tumors and lymphomas (Lyman et al., 2009; Pabst et al., 2012). hG‐CSF is also used in bone marrow transplantation (Khoury, 2006) and the treatment of severe congenital neutropenia (Rosenberg et al., 2006).

With the increased applications of hG‐CSF, attempts have been made to produce hG‐CSF using various hosts. However, recombinant hG‐CSF (rhG‐CSF), belonging to a group of glycoproteins, is produced in a nonglycosylated form by *Escherichia coli* (filgrastim/Neupogen®; Amgen), resulting in insoluble inclusion bodies, which require additional steps such as refolding (C. K. Kim et al., 2013; Mishra et al., 2020). rhG‐CSF in a glycosylated form produced from CHO cells (lenograstim/Granocyte®; Chugai Pharma) is available to treat specimens without further processing (M. O. Kim et al., 2006); however, the production process is time‐consuming. Using yeast as a host to produce hG‐CSF can increase the rate of production, while simultaneously producing the glycosylated form. Thus, attempts to produce hG‐CSF using yeast are still in progress, but the production level has not yet reached a commercial scale (Aggarwal & Mishra, 2020; Lasnik et al., 2001; Wittman et al., 2004).

To improve the production efficiency of rhG‐CSF from *S. cerevisiae*, we developed a secretion‐enhancing peptide cassette consisting of four parts: the *K. lactis* killer toxin secretion leader peptide, an hIL‐1β‐derived peptide, a peptide between the pro‐ and mature sequences of yeast mating factor‐α, and a kex2 proteolytic cleavage site. Among them, the effect of modifying synthetic propeptides containing an hIL‐1β‐derived peptide and yeast mating factor‐α was investigated in detail in batch cultures of *S. cerevisiae*. In particular, through a sequential three‐stage design and optimization involved in synthesized nascent protein folding and translocation, the production and secretion of heterologous protein rhG‐CSF from *S. cerevisiae* were significantly increased compared to the original synthetic propeptide, and protein recovery was enhanced.

## 2 MATERIALS AND METHODS

### 2.1 Design and construction of peptide vectors

**Figure 1:**
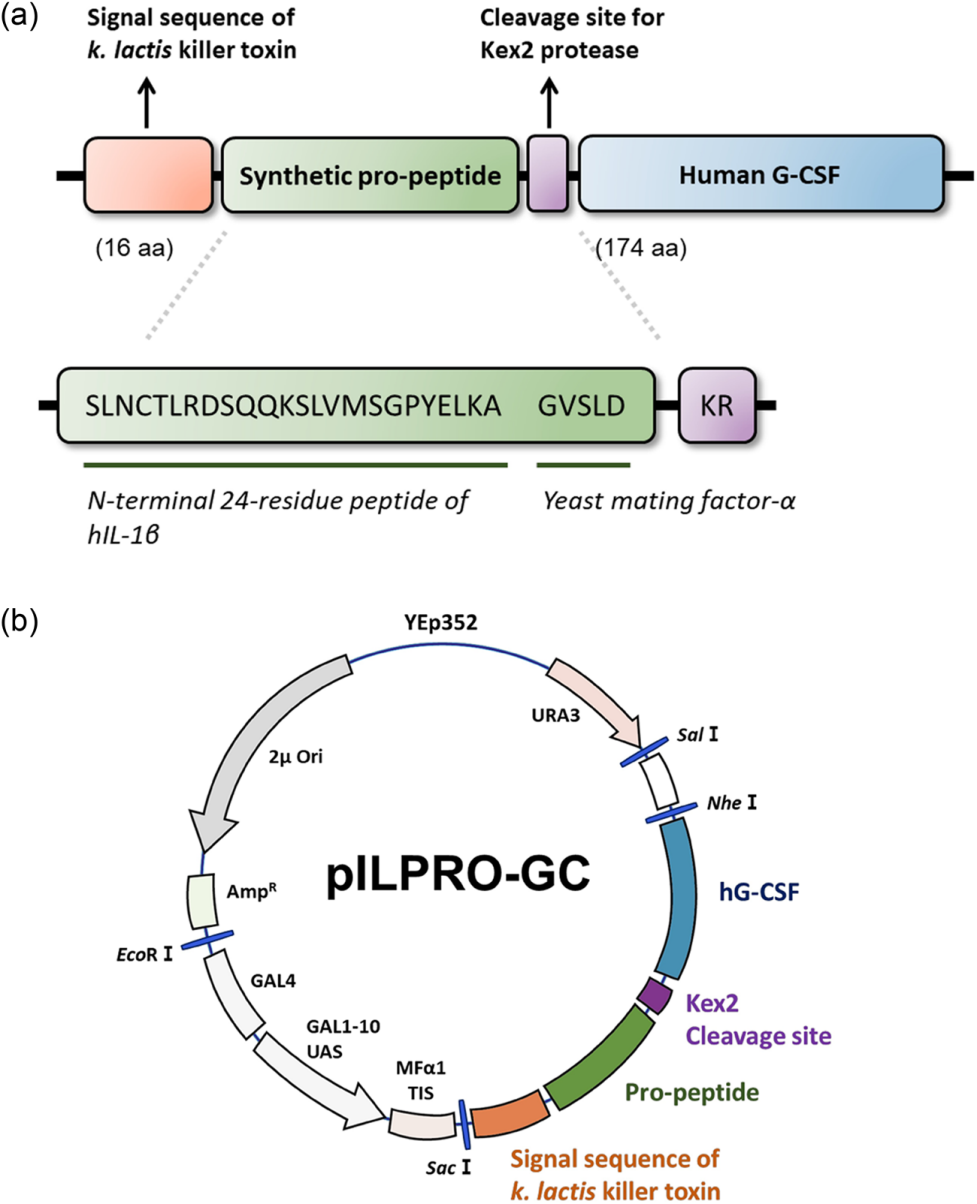
Schematic illustration of (a) secretion enhancing peptide cassette and (b) plasmid expression vector (pILPRO‐GC) (Ori, origin of replication; Amp^R^, ampicillin resistance gene; UAS, upstream activation sequence; YEp352, the original backbone of plasmid vector; MFα1 TIS, transcription initiation sequence of mating factor α1; *Eco*RI, *Sac*I, *Nhe*I, *Sal*I, restriction enzyme sites).

**Figure 2:**
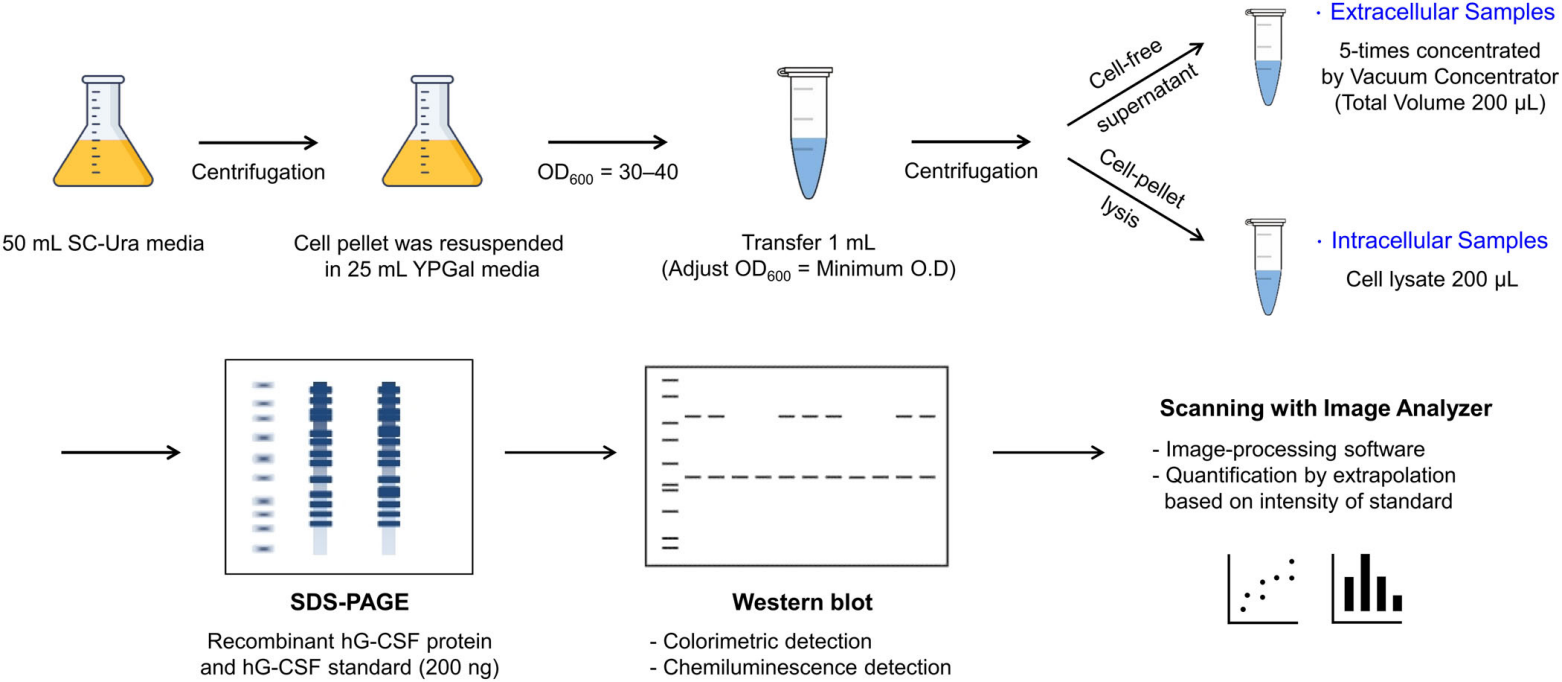
Schematic illustration of the experimental procedure of cell culture, sampling, detection, and quantification of recombinant human granulocyte colony‐stimulating factor (rhG‐CSF)

Clones at each stage were prepared using extension and fusion polymerase chain reaction (PCR) amplification using the appropriate primers, encoding the *NH*_2_‐*Sac*I‐killer toxin leader‐propeptide‐Kex2 cleavage site‐hG‐CSF‐*Nhe*I‐*COOH* (Figure 1a). All clones were sequentially ligated into the *Sac*I‐*Nhe*I site of the pIL20GC (Amp+) plasmid. The GAL1‐10 gene upstream activation sequence and the secretion leader sequence of the *K. lactis* killer toxin within the plasmid expression vector (pILPRO‐GC, Figure 1b) were cloned from the YEpsec. 1‐hIl plasmid vector (Baldari et al., 1987, Lee et al., 1999). Using standard recombinant DNA techniques for enzymatic manipulation of cloned DNA, plasmid expression vectors were constructed and modified, together with the stepwise sequence modification of the propeptide (Figure 2).

### 2.2 Transformation and selection

*S. cerevisiae* Y2805 (MATα pep4::HIS3, prb1‐Δ1.6R, can1, GAL2, his1‐200, ura3‐52) was grown in YPD (1% yeast extract, 2% bacto‐peptone, and 2% glucose), and transformed according to the Alkali‐Cation Yeast Transformation Kit manual (BIO 101 Inc.). The transformants were selected on SD‐Ura medium (0.8 g/L Complete Supplement Medium‐URA [MP Biomedical], 6.7 g/L yeast nitrogen base without amino acids [DIFCO: Becton Dickinson and Company], 2% glucose) agar plates after incubation for 3 days at 30°C.

### 2.3 Media and culture conditions

The transformed cells were cultivated overnight in a 3 ml SD‐Ura liquid medium (30°C, 250 rpm). The culture broth (1 ml) was inoculated in a 50 ml SD‐Ura liquid medium, followed by cultivation for 12 h at 30°C, and 210 rpm. The culture broth was centrifuged for 5 min at 2543*g*, and the cell pellet was resuspended in 25 ml YPGal medium (1% yeast extract, 2% bacto‐peptone, and 2% galactose) to induce recombinant gene expression using the GAL1‐10 upstream activating sequence, followed by a 24 h culture at 30°C.

### 2.4 Extraction of recombinant protein from *S. cerevisiae*

The yeast culture medium was monitored until the optical density of the culture at 600 nm (OD_600_) reached 30–40; further, the medium was diluted to 1 ml by adjusting the minimum OD to 30–40 for each stage, followed by centrifugation at 2390*g* for 10 min. The cell‐free supernatant was condensed fivefold using a vacuum concentrator (Ecospin 314; Biotron Inc.) before analysis of the extracellular recombinant proteins. The cell pellet was resuspended in 200 µl lysis buffer (200 mM Tris‐HCl, pH 7.9, 10 mM MgCl_2_, 1 mM EDTA, 5% glycerol, 1 mM dithiothreitol, 0.3 M ammonium sulfate, 1× protease inhibitor mix [Roche Diagnostics], and 1 mM PMSF). An equal volume of glass beads (425–600 µm; Sigma) was added, and the cells were vortexed and cooled on ice three times for 1 min each. After the cells were lysed, cell debris was removed by centrifugation at 16,200*g* for 10 min, and the cell lysate was analyzed to determine the intracellular recombinant protein content.

### 2.5 Analysis of secreted hG‐CSF

The recombinant hG‐CSF protein and hG‐CSF standard (Sigma) were subjected to 4%–12% sodium dodecyl sulfate‐polyacrylamide gel electrophoresis (4%–12% Bis‐Tris gel, Novex; Thermo Fisher Scientific Inc.), and transferred to a nitrocellulose membrane. The membrane was blocked with 5% skimmed milk in TBST buffer (Tris‐buffered saline [50 mM Tris‐HCl, pH 7.6, and 150 mM NaCl] with 1 mM Tween 20) at 25℃ for 1–2 h, followed by incubation with the primary antibody, mouse anti‐hG‐CSF immunoglobin G (IgG) (Santa Cruz Biotechnology) at 4°C for 16 h. The membrane was washed twice, for 10 min each, with TBST buffer and incubated with horseradish peroxidase‐conjugated antimouse secondary IgG (Santa Cruz Biotechnology) in TBST buffer for 1–2 h. After pouring off the TBST buffer, the membrane was reacted with an enhanced chemiluminescence solution (Thermo Fisher Scientific). The chemiluminescent bands were visualized using the Biomolecular Imaging System (LAS 3000 M; FujiFilm), and images were captured and analyzed using the image‐processing software Multi Gauge v2.3 (Fuji Film).

**Figure A1:**
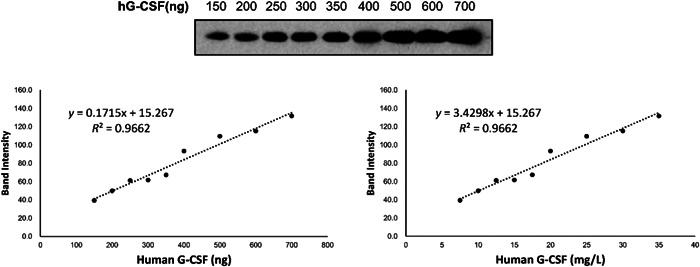
Standard curve for quantification of human granulocyte colony‐stimulating factor (hG‐CSF). Briefly, each commercial hG‐CSF in the range of 150–700 ng was prepared and was subjected to 4%–12% sodium dodecyl sulfate‐polyacrylamide gel electrophoresis (SDS‐PAGE). The proteins in the gel were transferred to a nitrocellulose membrane. For western blot analysis, mouse anti‐hG‐CSF antibodies (IgG) were used as primary antibodies, and the horseradish peroxidase‐conjugated goat anti‐mouse antibody was used as a secondary antibody. The band intensities of hG‐CSF standards were determined by scanning and analyzing the immunoreactive protein bands using a densitometer (GS‐800 Calibrated Densitometer; Bio‐Rad).

A standard curve in the range of 150–700 ng was prepared using a commercial hG‐CSF standard (Sigma), and rhG‐CSF was quantified by the chemiluminescent method (Figure A1). The immunoreactive protein bands were quantified through extrapolation based on the band intensity of the hG‐CSF standard (200 ng) loaded on each gel. The experimental procedure from culture to western blot analysis is shown schematically in Figure 2.

### 2.6 Enzymatic removal of *N*‐glyco‐moiety

The rhG‐CSF samples were denatured by boiling for 10 min at 100°C in 10× glycoprotein denaturing buffer (5% sodium dodecylsulfate, 0.4 M dithiothreitol) and treated with 10× G5 reaction buffer (0.5 M sodium acetate, pH 6.0) and endoglycosidase H (Endo H) (New England Biolabs Inc.) for 1h at 37°C. Samples treated with Endo H were confirmed by immunoblotting using a colorimetric method.

### 2.7 Calculations of the % increased efficiency of secretion

The % increased efficiency of secretion of the designed propeptides in each stage was calculated using the following equation:

$$\mathrm{The}\;\%\;\mathrm{increased}\;\mathrm{efficiency}\;\mathrm{of}\;\mathrm{secretion}=\frac{\mathrm{Extracellular}\;\mathrm{mature}\;\mathrm{rhG}–\mathrm{CSF}\;\mathrm{concentration}\;\mathrm{of}\;\mathrm{sample}}{\mathrm{Extracellular}\;\mathrm{mature}\;\mathrm{rhG}–\mathrm{CSF}\;\mathrm{concentration}\;\mathrm{of}\;\mathrm{control}(B)}\times 100.$$

## 3 RESULTS

### 3.1 Design of secretion enhancing peptide cassette for heterologous protein production from *S. cerevisiae*

To improve the secretion efficiency of hG‐CSF, we genetically fused the secretion‐enhanced peptide cassette containing hIL‐1β‐derived peptides upstream of the hG‐CSF gene (Figure 1a). The N‐terminus of hIL‐1β includes an *N*‐glycosylation site and can improve the secretion efficiency of target proteins by conjugating with *K. lactis* killer toxin secretion signal peptide (Choi et al., 2001; J. Lee et al., 2003; Song et al., 2002). The signal peptide of the *K. lactis* killer toxin is recognized and allowed to enter the ER, ensuring proper translocation of the expressed protein. The sequence Gly‐Val‐Ser‐Leu‐Asp (GVSLD), which is originally located between the pro‐ and mature sequences of yeast mating factor‐α, is known to enhance the efficiency of the Kex2 protease during cleavage of the C‐terminal side of NH_2_‐KR‐COOH (Rockwell et al., 2002). In summary, hG‐CSF was conjugated with the secretion‐enhancing peptide cassette, which consisted of the *K. lactis* killer toxin secretion leader peptide, followed by a 29‐residue propeptide (N‐terminal 24‐residue peptide [Ser5–Ala28] of hIL‐1β and GVSLD) and a Kex2 proteolytic cleavage site.

The process of transport from the ER to the Golgi apparatus in yeast is a rate‐limiting step in the secretory pathway of heterologous proteins (Bao et al., 2018; Hou et al., 2012; Van Zyl et al., 2016). Therefore, the secretion cassette was further designed and optimized to increase hG‐CSF folding in the ER, and consequently improve the efficiency of transport into the Golgi apparatus. The enhanced folding and transition of rhG‐CSF from the ER to the Golgi apparatus may significantly promote the secretion of rhG‐CSF into the extracellular medium.

**Figure 3:**
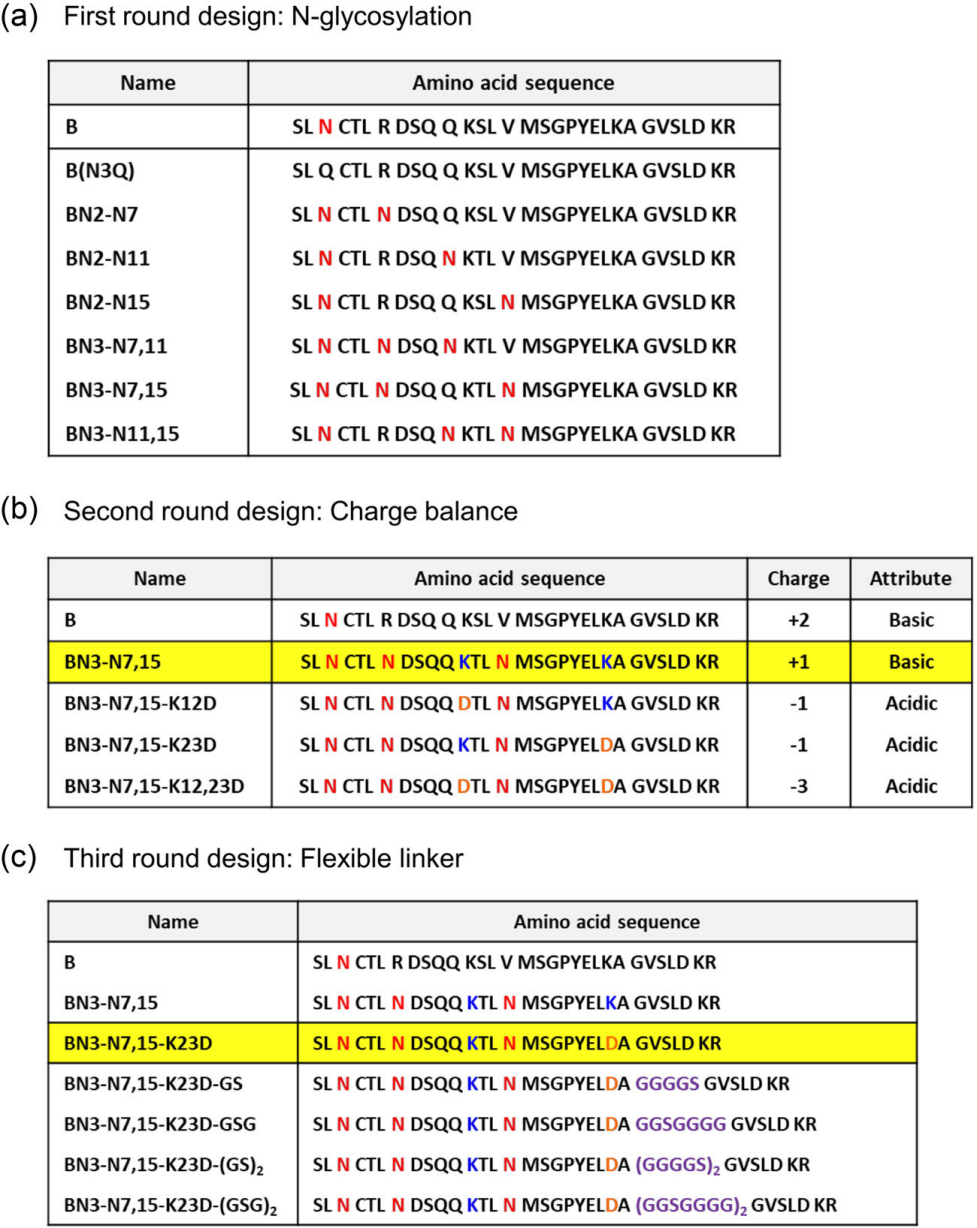
Propeptide sequences designed in the (a) first stage, (b) second stage, and (c) third stage (b, native sequence of original propeptide). The best propeptide sequence in the previous step is highlighted in yellow.

It has been hypothesized that the secretion of rhG‐CSF would be enhanced by *N*‐glycosylation, net charge balance, and insertion of a glycine‐rich flexible linker between hIL‐1β‐derived peptide and hG‐CSF (Figure 3). The effects of these three factors were investigated using a three‐stage screening and selection process. The concentrations of intracellular and extracellular rhG‐CSF were calculated using immunoblotting analysis at each stage of the propeptide sequence modification. Finally, the % increased efficiency of rhG‐CSF secretion was estimated as the ratio of the concentration of mature rhG‐CSF from the best propeptide sample in each stage to the concentration of mature rhG‐CSF from the original propeptide.

### 3.2 First stage design of the propeptide sequence: Inserting the *N*‐glycosylation site

*N*‐glycosylation of the propeptide derived from hIL‐1β, which occurs at a specific “Asn” site, has been found to play an important role in the secretion of heterologous proteins (K. S. Han et al., 2005; M. Han et al., 2014). *N*‐glycosylation plays a significant role in the secretion of hG‐CSF (K. S. Han et al., 2005), presumably because of the enhanced folding of rhG‐CSF in the ER (Doyon et al., 2002; Sagt et al., 2000), and is subsequently involved in the transition of rhG‐CSF from the ER to the Golgi apparatus (Ari et al., 2001).

**Figure A2:**
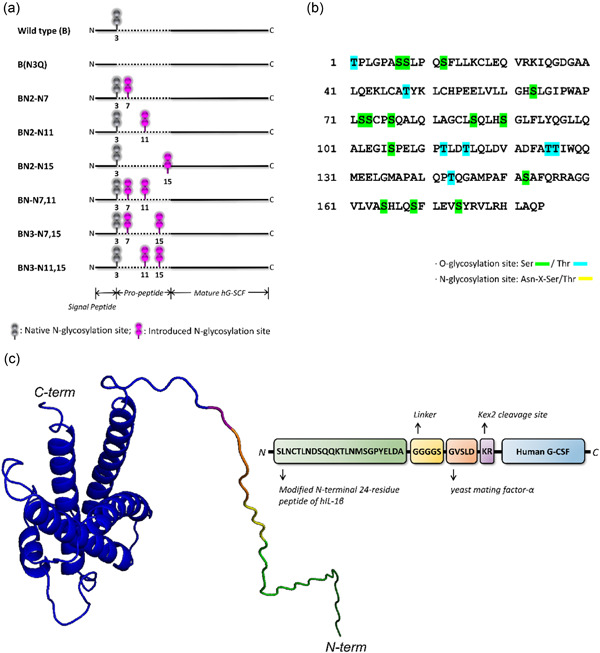
(a) Introduction of *N*‐glycosylation sites to the synthetic propeptide. (b) An amino acid sequence of the human granulocyte colony‐stimulating factor (hG‐CSF) protein marked with possible glycosylation sites. (c) The predicted 3D structure of hG‐CSF fused to the N‐terminus with a secretion‐enhancing peptide cassette (BN3‐N7,15‐K23D‐GS in the third stage) was rendered using PyMOL with coordinates obtained from the RCSB Protein Data Bank (1GNC for hG‐CSF). The color of each block in the vector design matches the color in the structure.

Therefore, we inserted up to three *N*‐glycosylation sites by replacing Lys7 with Asn7, Gln11 with Asn11, and Val15 with Asn15 to produce Asn–X–Thr/Ser. As shown in Figures 3a and A2a, we implemented six changes in the propeptide sequence (BN2‐N7, ‐N11, ‐N15, BN3‐N7,11, ‐N7,15, ‐N11,15). We also removed an original propeptide “B” *N*‐glycosylation site by switching Asn3 to Gln3 to confirm the effect of *N*‐glycosylation (see peptide B[N3Q] in Figure 3a). Thus, we designed seven propeptides in the first stage: B(N3Q), BN2‐N7, ‐N11, ‐N15, BN3‐N7,11, ‐N7,15, ‐N11,15. Mature rhG‐CSF had a confirmed molecular weight (MW) of 18.6 kDa and *N*‐glycosylation of the rhG‐CSF was confirmed by deglycosylation of the extracellular and intracellular samples using the Endo H enzyme, which cleaves between tandem N‐acetyl glucosamines.

**Figure 4:**
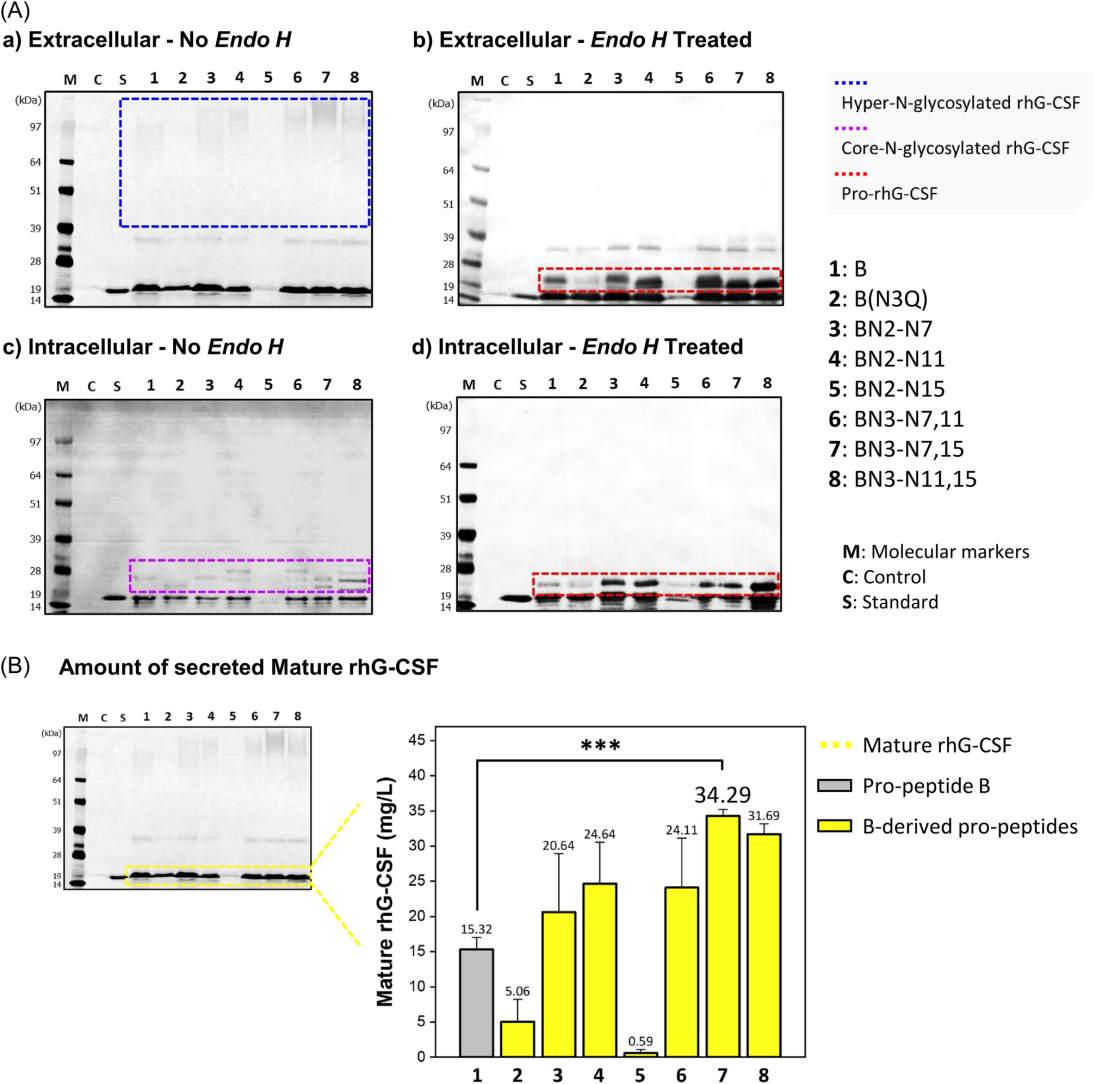
(A) Representative image of western blot analyses of extracellular (a, b) and intracellular (c, d) samples from *Saccharomyces cerevisiae* cultures that express recombinant human granulocyte colony‐stimulating factor (rhG‐CSF) using the propeptides designed in the first stage. Western blot results of *Endo H*‐treated extracellular and intracellular samples are also shown in b and d, respectively. (Standard, the commercial standard of human granulocyte colony‐stimulating factor [hG‐CSF]; Control, samples from the culture of *S. cerevisiae* Y2805; B, original propeptide; B[N3Q] to BN3‐N11,15, B‐derived propeptides designed in the first stage). (B) The chemiluminescent bands (extracellular mature rhG‐CSF) were quantified through extrapolation based on the band intensity of the standard. Each bar represents the average with the error bars indicating standard deviations (*n* = 3). The *p*‐values were analyzed by Student's *t*‐test (***p < 0.001).

**Figure A3:**
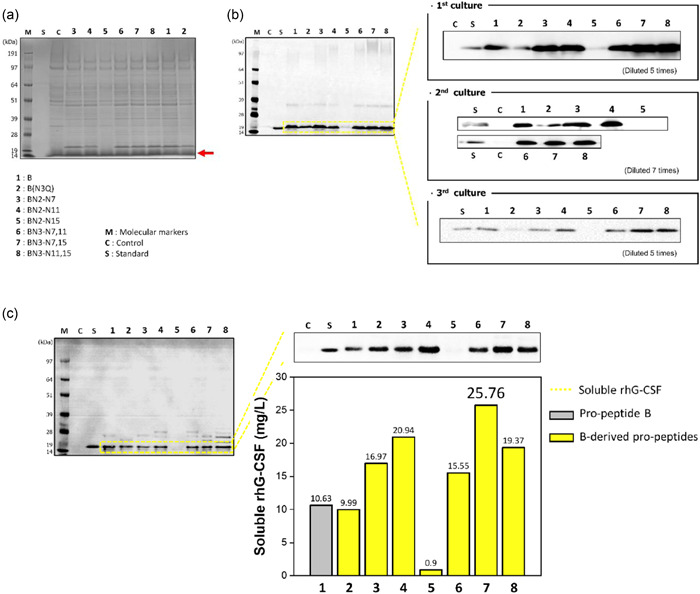
(a) Sodium dodecyl sulfate‐polyacrylamide gel electrophoresis (SDS‐PAGE) analysis of extracellular recombinant human granulocyte colony‐stimulating factor (rhG‐CSF) from the first stage, stained with Coomassie Brilliant Blue. The red arrow indicates mature rhG‐CSF (18.6 kDa). (b) The chemiluminescent visualization of western blot analysis results of extracellular mature rhG‐CSF from the first stage. (c) The chemiluminescent bands (intracellular soluble rhG‐CSF) were quantified through extrapolation based on the band intensity of the standard.

Recombinant proteins, except for BN2‐N15, were detected at approximately 18.6 kDa for mature rhG‐CSF in intracellular and extracellular samples (Figures 4 and A3), indicating that all proteins except BN2‐N15 were successfully expressed and secreted by *S. cerevisiae*. Of the extracellular proteins, B(N3Q), without *N*‐glycosylation sites, did not show hyper‐*N*‐glycosylated proteins (high MW proteins within the blue dotted box); however, the other proteins with *N*‐glycosylation sites were observed in hyper‐*N*‐glycosylated forms with mature rhG‐CSF. Of the intracellular proteins, the core‐*N*‐glycosylated proteins (low‐MW proteins in the purple dotted box) were observed at various sizes below 28 kDa in most of the samples. After Endo H treatment, all proteins except for B(N3Q) and BN2‐N15 were converted to low‐MW proteins (pro‐rhG‐CSF; 23 kDa) within the red dotted box, showing increased band thickness with the insertion of the *N*‐glycosylation site. The band that is still visible between 28 and 39 kDa in the extracellular product after enzyme treatment is presumed to be the result of *O*‐glycosylation in the propeptides or protein (Figure A2b).

As shown in Figure 4B, the ratio of extracellular mature rhG‐CSF on original propeptide B to BN3‐N7,15 was approximately two‐fold, but compared to B(N3Q) had an approximately 0.3‐fold expression level, indicating that *N*‐glycosylation plays a key role in rhG‐CSF secretion. BN3‐N7,15 showed the highest expression of 34.29 mg/L. Overall, we observed that a large amount of rhG‐CSF in both mature and hyper‐*N*‐glycosylated forms was secreted into the YPGAL medium through first‐stage optimization.

### 3.3 Second stage design of the propeptide sequence: Substituting a positive net charge for a negative or neutral net charge in the propeptide sequence

In the second stage, we substituted amino acids with a positive charge (Lys, K) for amino acids with a negative charge (Asp, D), based on the best propeptide from the first stage (BN3‐N7,15). Johansson et al. (1993) and Kajava et al. (2000) reported that the charge balance between the N‐terminus of the signal peptide and the mature moiety is important for the secretion efficiency of gram‐negative bacteria (Johansson et al., 1993; Kajava et al., 2000). In addition, acidic and neutral propeptides are known to be effective in enhancing the secretion efficiency of heterologous proteins in gram‐positive bacteria (Le Loir et al., 2001). Similar research has been conducted on yeast, but the exact causal relationship has not yet been elucidated (Liang et al., 2013; Massahi & Çalık, 2016; Yarimizu et al., 2015).

**Figure 5:**
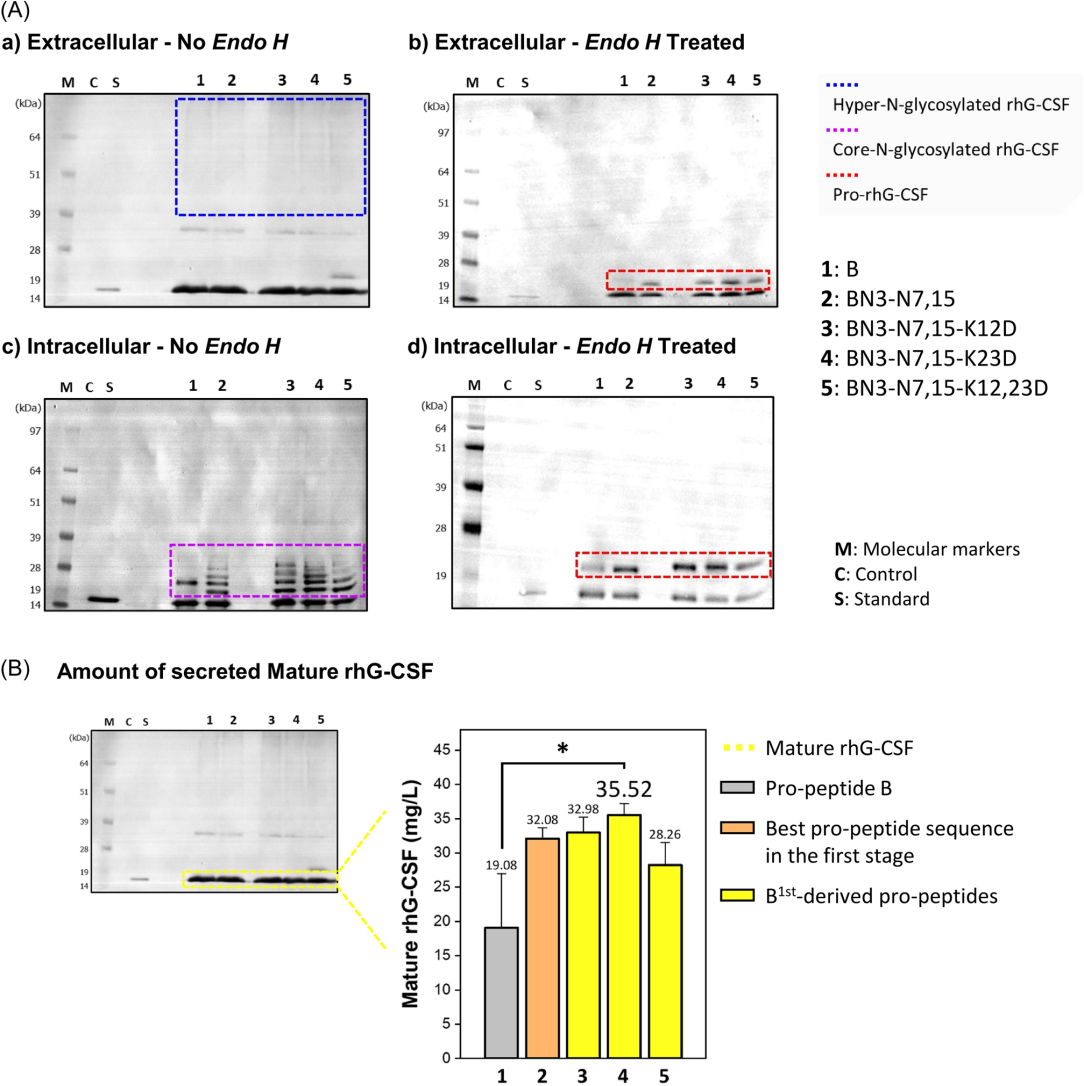
(A) Representative image of western blot analyses of extracellular (a, b) and intracellular (c, d) samples from *Saccharomyces cerevisiae* cultures that express recombinant human granulocyte colony‐stimulating factor (rhG‐CSF) using the propeptides designed in the second stage. Western blot results of *Endo H*‐treated extracellular and intracellular samples are also shown in b and d, respectively (Standard, the commercial standard of human granulocyte colony‐stimulating factor [hG‐CSF]; Control, samples from the culture of *S. cerevisiae* Y2805; B, original propeptide; BN3‐N7,15, best propeptide sequence in the first stage; BN3‐N7,15‐K12D to BN3‐N7,15‐K12,23D, BN3‐N7,15‐derived propeptides designed in the second stage). (B) The chemiluminescent bands (extracellular mature rhG‐CSF) were quantified through extrapolation based on the band intensity of the standard. Each bar represents the average with the error bars indicating standard deviations (*n* = 3). The *p‐*values were analyzed by Student's *t*‐test (*p < 0.05).

**Figure A4:**
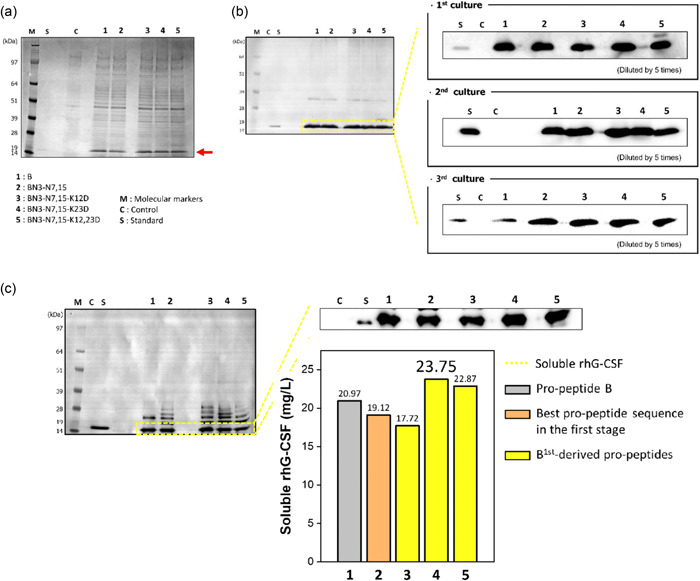
(a) Sodium dodecyl sulfate‐polyacrylamide gel electrophoresis (SDS‐PAGE) result of extracellular recombinant human granulocyte colony‐stimulating factor (rhG‐CSF) from the second stage, stained with Coomassie Brilliant Blue. The red arrow indicates mature rhG‐CSF (18.6 kDa). (b) The chemiluminescent visualization of western blot analysis results of extracellular mature rhG‐CSF from the second stage. (c) The chemiluminescent bands (intracellular soluble rhG‐CSF) were quantified through extrapolation based on the band intensity of the standard.

In this study, all the propeptides used in the first stage had a net positive charge. Therefore, we investigated whether replacing the net positive charge with a net negative or neutral charge in the propeptide could improve the secretion efficiency. Especially, given that the aspartic acid of the protein affects solubility (Trevino et al., 2007), we replaced lysine at sites 12 and 23 in the BN3‐N7,15 propeptide, with aspartic acids (Figure 3b). Consequentially, in the second stage, all the recombinant proteins with acidic propeptides were successfully expressed and secreted from *S. cerevisiae* (Figures 5 and A4). The hyper‐*N*‐glycosylated extracellular proteins within the blue dotted box and the core *N*‐glycosylated proteins within the purple dotted box were converted into pro‐rhG‐CSF by Endo H treatment.

The various bands of intracellular proteins within the purple dotted box represent the core *N*‐glycosylated proteins in the ER, and the core *N*‐glycosylated protein patterns differed depending on the increase in *N*‐glycosylation sites, although the net charge did not appear to have any significant effect. The concentrations of secreted rhG‐CSF in *S. cerevisiae* also did not increase dramatically (Figure 5B), unlike the high secretion efficiency observed in bacteria such as *E. coli*, indicating that secretion in *S. cerevisiae* was not significantly affected by the negative charge of the propeptide in this experiment. When compared to the first‐step best peptide, an improvement of only 15% was observed, which was not significant. The effect of secretion by charge balance of the secretion‐enhancing peptide cassette on eukaryotic secretion is greatly affected by external factors, such as host or target protein. Thus, secretion can be considerably different depending on the target protein, which is vulnerable to the charge balance (Massahi & Çalık, 2016). However, despite no drastic enhancement in secretion observed in the first stage, the amount of extracellular mature rhG‐CSF utilizing BN3‐N7,15‐K23D increased slightly with the propeptides compared to those utilizing BN3‐N7,15.

### 3.4 Third stage design of the propeptide sequence: Addition of a flexible linker sequence between the propeptide and hG‐CSF

Propeptide BN3‐N7,15‐K23D showed the best performance in the second stage in enhancing the secretion of mature rhG‐CSF. In the third stage, four additional propeptides were designed by inserting an appropriate linker in the region between the hIL‐1β‐derived peptide and hG‐CSF. Flexible glycine‐rich regions have been known to be natural linkers in proteins, as they consist of loops that connect domains in multi‐domain proteins (Reddy Chichili et al., 2013). A GS linker was earlier inserted between GM‐CSF and interleukin (IL)‐3 to provide conformational flexibility, and consequently enhance the folding of GM‐CSF and IL‐3 to their native form (Curtis et al., 1991; A. Y. Lee et al., 2003).

In the third stage, we inserted four types of glycine/serine linkers into the best second‐stage sequence, BN3‐N7,15‐K23D. The propeptides BN3‐N7,15‐K23D‐GS/‐GSG/‐(GS)_2_/‐(GSG)_2_ were prepared using extension PCR and inserted before GVSLD in BN3‐N7,15‐K23D (Figure 3c). The design of these propeptides incorporates a flexible linker sequence, maximizing the opportunity for hG‐CSF and the propeptide derived from IL‐1β to fold into their native three‐dimensional structures and become functionally independent (Figure A2c).

**Figure 6:**
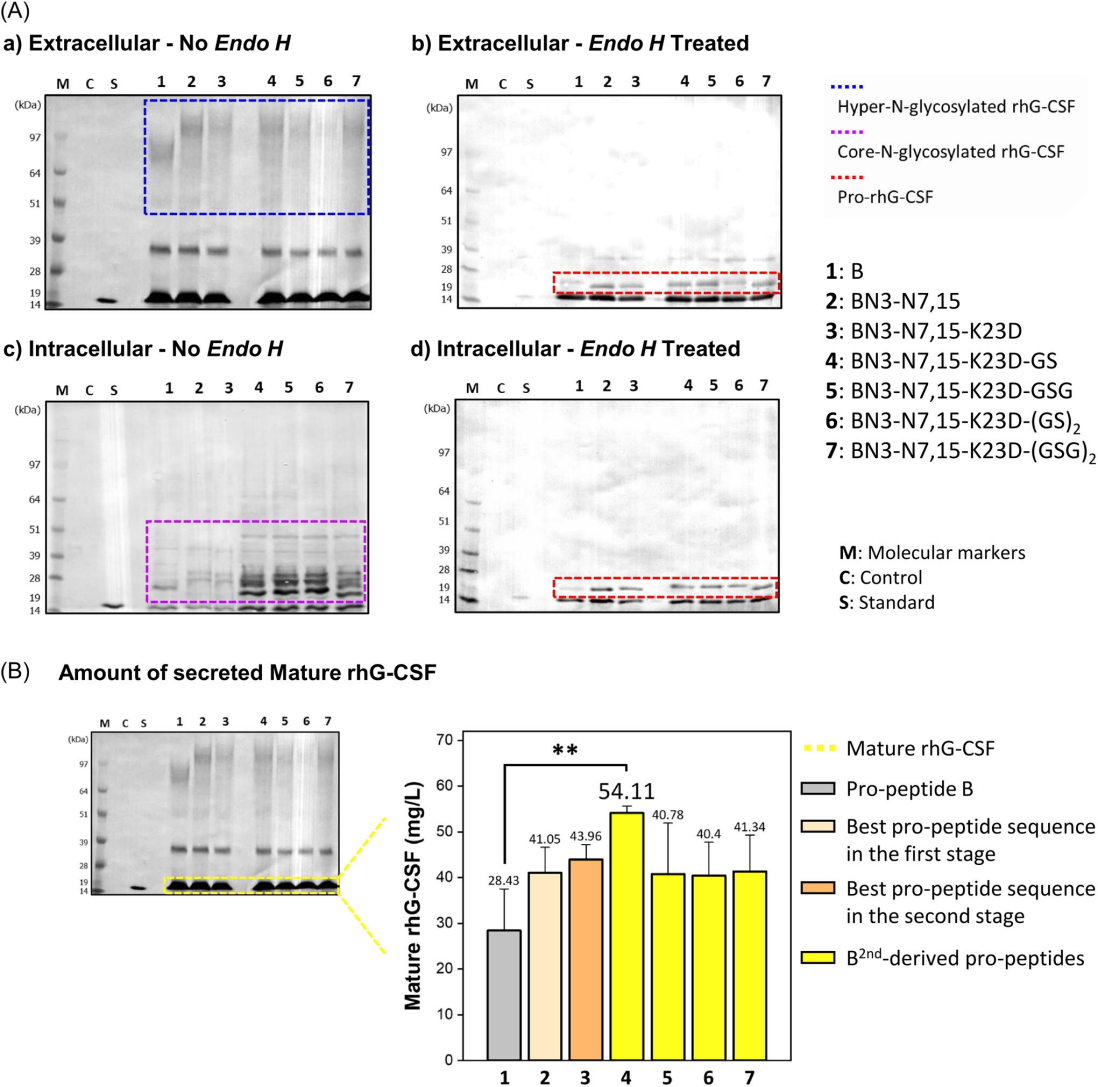
(A) Representative image of western blot analyses of extracellular (a, b) and intracellular (c, d) samples from *Saccharomyces cerevisiae* cultures that express recombinant human granulocyte colony‐stimulating factor (rhG‐CSF) using the propeptides designed in the third stage. Western blot results of *Endo H*‐treated extracellular and intracellular samples are also shown in b and d, respectively (Standard, the commercial standard of human granulocyte colony‐stimulating factor [hG‐CSF]; Control, samples from the culture of *S. cerevisiae* Y280; B, original propeptide; BN3‐N7,15, best propeptide sequence in the first stage; BN3‐N7,15‐K23D, best propeptide sequence in the second stage; BN3‐N7,15‐K23D‐GS to BN3‐N7,15‐K23D‐(GSG)_2_; BN3‐N7,15‐K23D‐derived propeptides designed in the third stage). (B) The chemiluminescent bands (extracellular mature rhG‐CSF) were quantified through extrapolation based on the band intensity of the standard. Each bar represents the average with the error bars indicating standard deviations (*n* = 3). The *p*‐values were analyzed by Student's *t*‐test (**p < 0.001).

**Figure A5:**
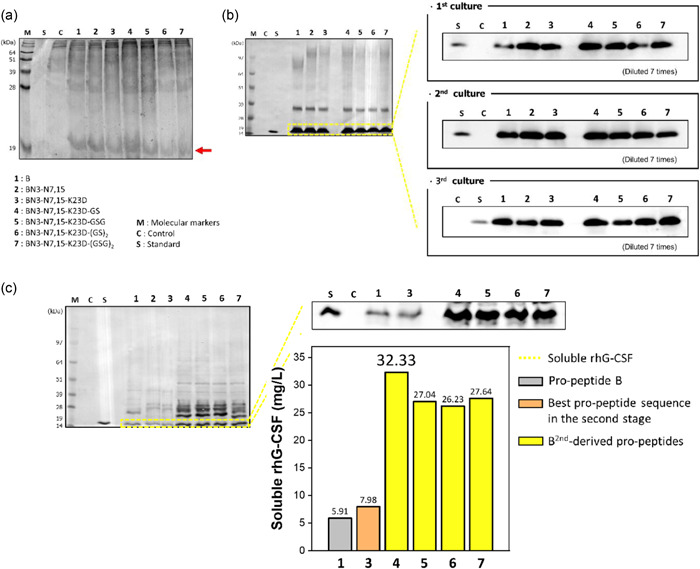
(a) Sodium dodecyl sulfate‐polyacrylamide gel electrophoresis (SDS‐PAGE) result of extracellular recombinant human granulocyte colony‐stimulating factor (rhG‐CSF) from the third stage, stained with Coomassie Brilliant Blue. The red arrow indicates mature rhG‐CSF (18.6 kDa). (b) Chemiluminescent visualization of western blot analysis results of extracellular mature rhG‐CSF from the third stage. (c) The chemiluminescent bands (intracellular soluble rhG‐CSF) were quantified through extrapolation based on the band intensity of the standard.

As shown in Figures 6 and A5, both extracellular hyper‐*N*‐glycosylated (within the blue dotted box) and mature rhG‐CSF were detected by western blot analysis. The intracellular proteins also showed core *N*‐glycosylation (within the purple dotted box) and mature rhG‐CSF. After Endo H treatment, the hyper‐*N*‐glycosylated and core *N*‐glycosylated hG‐CSFs were completely converted to pro‐hG‐CSF, as shown in the red dotted box.

In the third stage, except for the propeptide with the ‐GS linker, the other propeptides with linkers did not show a dramatic enhancement in secretion, compared to the best second‐stage pro‐peptides. BN3‐N7,15‐K23D with a GS linker showed the highest secretion efficiency for extracellular mature hG‐CSF. Consequently, through a sequential three‐stage design screening, selection, and optimization, using BN3‐N7,15‐K23D‐GS, the amount of extracellular mature rhG‐CSF increased from 28.427 mg/L (before the first stage) to 54.106 mg/L (after the third stage) in a 25‐ml flask culture, and the % increased efficiency of secretion was 190% higher than that of the initial sequence of propeptide “B” (Table 1).

**Table 1:** The % increased efficiency of secretion of mature recombinant human granulocyte colony‐stimulating factor (rhG‐CSF) using the best propeptides at each optimization stage

	Extracellular mature rhG‐CSF (mg/L)	% Increased efficiency of secretion
B	28.43 ± 9.09	100
BN3‐N7,15	41.05 ± 5.61	144
BN3‐N7,15‐K23D	43.96 ± 3.29	155
BN3‐N7,15‐K23D‐GS	54.11 ± 1.54	190

## 4 DISCUSSION

Heterologous protein production using *S. cerevisiae* has many advantages, including the secretion of proteins with post‐translational processing. Although large‐scale production and downstream processing of some proteins expressed in *S. cerevisiae* are well‐established procedures, the secretion efficiency of *S. cerevisiae* and the conditions necessary for heterologous protein production are sometimes highly specific to the target protein and the expression system. Therefore, the system should be tailored for high‐level production.

The design and optimization of signal/leader peptides is a relatively simple and efficient strategy that can be easily tuned for the high‐level production of many target proteins. The hIL‐1β‐derived peptide (Ser5 to Ala28) fused to the N‐terminus of target proteins such as cytokines, hormones, enzymes, or viral surface antigens, in conjunction with an N‐terminal secretion signal, improves the secretion of target proteins by *S. cerevisiae* (Choi et al., 2001; J. Lee et al., 2003; Song et al., 2002). The reason for the improved secretion of hIL‐1β‐derived peptide‐linked heterologous recombinant proteins is not clear. The transport of proteins from the ER to the Golgi apparatus is a rate‐limiting step that affects the secretion efficiency in yeast. The ER is equipped with relevant factors for proper protein folding (e.g., molecular chaperones—BiP, calnexin, and calreticulin and foldase—protein disulfide isomerase) and the removal of misfolded proteins (e.g., ER‐associated degradation) (Brodsky & Skach, 2011; Celik & Calik, 2012). However, because these factors are also present in limited quantities, they are insufficient to accommodate all misfolded proteins. Eventually, misfolded proteins accumulate in the ER, preventing further movement along the secretory pathway. Thus, we presumed that the peptide plays an important role in enhancing target protein solubility and folding in the ER, and further in target transport to the Golgi apparatus.

However, as linking the hIL‐1β propeptide does not always assure identically superior secretion efficiencies of many target proteins, a rational strategy is required to find the optimal propeptide for maximally enhancing the target protein folding and translocation for secretion of a target protein.

In this study, the strategy for heterologous protein secretory expression focuses mainly on propeptide engineering to enhance the solubility of heterologous proteins, and consequently improve the efficiency of their transport from the ER to the Golgi apparatus. Beginning with the N‐terminal peptide (Ser5 to Ala28) of the original hIL‐1β, we designed various propeptides for the secretion of hG‐CSF, the model heterologous protein in *S. cerevisiae*, and inserted them between the secretion leader peptide of the *K. lactis* killer toxin and hG‐CSF. Stepwise sequence optimization of the propeptides was performed by estimating the following parameters: the amount of intracellular soluble rhG‐CSF, amount of extracellular mature rhG‐CSF, and extent of secretion of rhG‐CSF. In the first stage of the design of the propeptide, *N*‐glycosylation was induced by inserting “Asn” at various sites in the sequence (Figure 3a). We hypothesized that *N*‐glycosylation could enhance protein secretion and our results were consistent with this. The amount of secreted mature rhG‐CSF of the propeptide [BN3‐N7,15], which induces the most *N*‐glycosylation, was almost seven times higher than that of the propeptide [B(N3Q)] without *N*‐glycosylation. Therefore, the *N*‐glycosylation of the propeptide plays a key role in enhancing the secretion of hG‐CSF, presumably because of enhanced protein folding and trafficking through the ER and Golgi apparatus. However, among the various trials, *N*‐glycosylation did not always enhance protein secretion. Even if only the position of the *N*‐glycosylation site in the best first‐stage propeptide sequence was changed, hG‐CSF secretion was almost completely inhibited. This shows that depending on the site of N‐glycosylation, it may have interfered with folding and secretion (Ellgaard & Helenius, 2003; Sagt et al., 2000).

In the second stage, given that the net negative charge of the propeptide affects protein solubility (Trevino et al., 2007), the propeptide associated with hG‐CSF was designed with a net negative charge and contributed to an increase in the synthesis of total (pro‐ and mature) rhG‐CSF. However, the secretion efficiency from *S. cerevisiae* did not increase drastically, unlike the high secretion efficiency observed in prokaryotic expression systems, such as *E. coli*, indicating that secretion in eukaryotes was not significantly affected in this experiment.

In the third stage, the propeptide design incorporated a flexible linker sequence to maximize the opportunity for rhG‐CSF and the propeptide derived from IL‐1β to fold into their native three‐dimensional structures. The amount of extracellular mature rhG‐CSF increased considerably when conformational flexibility was added to the propeptide by inserting internal glycine/serine residues. When the flexible GS linker was inserted into the propeptide, the expression of extracellular mature rhG‐CSF reached a maximum of 54.1 mg/L, which was 1.9‐fold greater than the initial propeptide “B” (Table 1). This value is higher than that reported in other studies (yields up to approximately 39 mg/L) in which rhG‐CSF was produced using the α‐MAT secretion signal in another yeast species, *Pichia pastoris* (Aggarwal & Mishra, 2020; Lasnik et al., 2001).

In summary, through a three‐stage design involving screening, selection, and optimization, the production and secretion of rhG‐SCF from *S. cerevisiae* substantially increased compared to the original propeptide, resulting in an enhanced protein recovery yield. Although rhG‐CSF was used as the model protein in this study, this stepwise rational design of a propeptide is a promising strategy for enhancing the secretion of other heterologous proteins, using *S. cerevisiae* as the host.

## AUTHOR CONTRIBUTIONS

**Ji Sung Cho**: Investigation (equal); writing—original draft (lead). **Hye Ji Oh**: formal analysis (equal); investigation (equal). **Young Eun Jang**: formal analysis (equal); investigation (equal); writing—review & editing (lead). **Hyun Jin Kim**: formal analysis (equal). **Areum Kim**: writing—original draft (supporting). **Jong‐Am Song**: conceptualization (supporting). **Eun Jung Lee**: supervision (equal); writing—review & editing (lead). **Jeewon Lee**: conceptualization (lead); supervision (lead); writing—review & editing (equal).

## CONFLICT OF INTEREST

None declared.

## ETHICS STATEMENT

Work with recombinant DNA and microorganisms was performed according to national requirements.

## Data Availability

All data are provided in full in the results section of the paper. The DNA sequence encompassing the human granulocyte colony‐stimulating factor (hG‐CSF) is available at www.ncbi.nlm.nih.gov/nuccore/NM_172219

## APPENDIX A

See Figures A1, A2, A3, A4, A5.
